# Revealing the Impact of Gel Electrolytes on the Performance of Organic Electrochemical Transistors

**DOI:** 10.3390/gels11030202

**Published:** 2025-03-14

**Authors:** Mancheng Li, Xiaoci Liang, Chuan Liu, Songjia Han

**Affiliations:** 1 State Key Laboratory of Optoelectronic Materials and Technologies, Guangdong Province Key Laboratory of Display Material and Technology, School of Electronics and Information Technology, Sun Yat-Sen University, Guangzhou 510275, China; limch27@mail2.sysu.edu.cn (M.L.); liangxc5@mail.sysu.edu.cn (X.L.); liuchuan5@mail.sysu.edu.cn (C.L.); 2 College of Electronic Engineering, College of Artificial Intelligence, South China Agricultural University, Guangzhou 510642, China

**Keywords:** gel electrolyte, organic electrochemical transistors, capacitance, mechanisms

## Abstract

Gel electrolyte-gated organic electrochemical transistors (OECTs) are promising bioelectronic devices known for their high transconductance, low operating voltage, and integration with biological systems. Despite extensive research on the performance of OECTs, a precise model defining the dependence of OECT performance on gel electrolytes is still lacking. In this work, we refine the device model to comprehensively account for the electrical double layer (EDL)’s capacitance of the gel electrolyte. Both experimental data and theoretical calculations indicate that the maximum transconductance of the OECT is contingent upon ion concentration, drain voltage, and scan rate, highlighting a strong correlation between the transconductance and the hydrogel electrolyte. Overall, this model serves as a theoretical tool for improving the performance of OECTs, enabling the further development of bioelectronic devices.

## 1. Introduction

Gels, due to their mechanical flexibility, biocompatibility [1], and ionic conductivity [2], are widely applied in areas such as drug delivery [3], tissue engineering [4], pressure sensors [5], and bioelectronics [6]. Among them, solid-state gels exhibit distinct advantages over their liquid-state counterparts in terms of device integration and operational stability [7]. Recent advances have focused on ion gels and hydrogels, which combine solid structure, soft nature, superior mechanical properties and ionic conductivity, making them ideal electrolytes for advanced bioelectronic devices, like organic electrochemical transistors (OECTs) [8]. OECTs offer advantages such as simple structure, high sensitivity, and low operating voltage [9,10], making them suitable for applications like electro-physiological signal recording [11], ion sensing [12], and artificial synapses [13,14]. Unlike traditional field-effect transistors (FETs), OECTs use organic mixed ionic–electronic conductors as the channel and ion-rich hydrogels as the electrolyte layer. For instance, glycerol-enhanced polyvinyl alcohol (PVA) hydrogel-gated transistors have demonstrated superior electrical stability, longevity, and flexibility [7]. Moreover, PVA organogel-gated vertical OECTs exhibit higher ionic conductivity, enhanced transconductance, and faster switching speed compared to ion gel-gated and planar devices [15]. While these attributes make OECTs highly promising for bioelectronics, optimizing their performance in advanced applications requires a deeper understanding of how the hydrogel electrolyte affects OECTs’ operating mechanism and electrochemical performance.

A classical operation model of OECTs was proposed by Bernards and Malliaras [16], describing these devices as consisting of two circuits: one electronic and one ionic. The ionic circuit is represented by an equivalent circuit of a resistor in series with a capacitor. Subsequently, several approaches have been proposed to extend the Bernards model by introducing parameters that account for OECT-specific properties [17,18,19,20]. For example, Paudel et al. proposed key factors such as drain bias, channel thickness, and length, which influence peak transconductance [21]. V. Kaphle et al. used a 2D drift-diffusion model to reveal ion distribution in the transistor channel and its impact on device performance [20]. A. Weissbach et al. investigated the impact of electrochemical electrode coupling, gate electrode, and electrode/channel overlap on OECT performance, revealing how these factors affected output saturation, threshold voltage, and circuit optimization [22]. However, a model that accounts for the nonlinear effects induced by the electrical double layer (EDL) of the hydrogel electrolyte, as well as the electrochemical reactions triggered by ion injection, needs to be proposed. Therefore, we specifically focus on understanding the impact of the EDL effect and ionic dynamics on the electrochemical properties of OECTs.

In this study, we present an improved two-dimensional description model for OECTs that incorporates the electric double-layer (EDL) effect of the gel electrolyte. By considering the EDL, we accurately characterize the ion distribution within the gel electrolyte under varying ion concentration, drain potential, and scan rate. Subsequently, through experiments, we identify and validate the influence of the hydrogel electrolyte on the transconductance. The trend in transconductance variation observed in the experimental measurement results shows great agreement with that predicted by our model, which not only validates the accuracy of our model but also provides strong evidence for the understanding of the underlying mechanisms governing the transconductance behavior of OECTs in relation to gel electrolytes. Therefore, we propose that this improved model has the potential to guide the design of more efficient OECTs and facilitate performance optimization, opening up new possibilities for their applications in various fields, such as bio-sensing, flexible electronics, and energy storage devices.

## 2. Results and Discussion

### 2.1. Analysis of the Operating Mechanism of Gel-Based OECTs

**Figure 1:**
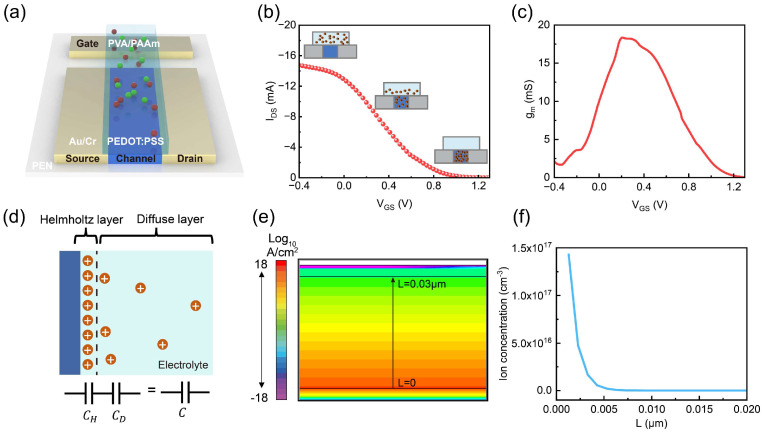
(**a**) Schematic of OECT (with Na⁺ ions (red balls) and Cl⁻ ions (green balls) in the hydrogel). (**b**) Transfer curve of OECT. (**c**) Transconductance curve of OECT. (**d**) Schematic and capacitance model of the electrolyte. (**e**) Ion distribution simulation by Silvaco TACD. (**f**) Ion concentration distribution diagram from the vertical cutline in (**e**).

Figure 1a illustrates the schematic of a typical gel electrolyte-gated OECT, featuring coplanar source, drain, and gate electrodes, a semiconducting channel, and a hydrogel electrolyte. Unlike traditional field-effect transistors (FETs), which operate based on the field-effect mechanism (Figure S1a), organic electrochemical transistors (OECTs) function through electrochemical reactions (Figure S1b). The working mechanism of OECTs relies on the gate voltage controlling the injection of ions from the electrolyte into the organic semiconductor channel to undergo electrochemical reactions, as shown in Figure S1c [23].

The preparation process of the dual-network hydrogel electrolyte is shown in Figure S2. The monomer AM crosslinks with MBA under the activation of the photoinitiator, forming PAAm, which, together with PVA, forms a double-network hydrogel. EG and Gly primarily function to improve the hydrogel’s water retention, while Na^+^ serves as free-moving ions in the hydrogel electrolyte. Figure 1b shows the transfer curve of the gel electrolyte-gated OECT. When a positive voltage is applied, the cations (Na⁺) in the electrolyte gradually migrate and accumulate on the semiconductor surface, forming a double layer. Owing to the high potential between the gate and channel layers, lots of Na⁺ ions gradually migrate to the PEDOT:PSS film. Therefore, they undergo complexation reactions with PSS chains, reducing the conductive PEDOT⁺ to non-conductive PEDOT⁰. This leads to a decrease in the transistor channel current. Additionally, as the gate potential continues to rise, an increasing number of ions are injected into the semiconductor channel. Consequently, the channel current decreases more markedly, demonstrating the transfer characteristic of a depletion-mode transistor. Here, the property of the OECT is valued by transconductance ($g_{m}$, Equation (1)), which is the slope of the transfer curves and describes the inherent amplification of the OECT, corresponding to the ability of the device to transduce ionic signals into electrical signals (Figure 1c). We defined the curve peak as the maximum transconductance ($g_{m,\;max}$), and the corresponding gate voltage was denoted as $V_{G,\;max}$.

Differently from traditional FETs, whose transconductance $g_{m}$ is independent of the gate potential in the linear region and linear with the gate potential in the saturation region, in OECTs, the experimental results reveal a strong non-monotonic dependence of transconductance on gate potential, the transconductance exhibits a bell-shaped behavior, and a distinct peak transconductance $g_{m,\;max}$ is usually found [24,25,26]. To better understand the reason for the peak in transconductance and establish a design methodology of OECTs, the 2D drift-diffusion simulation was first described [16,27,28]. As shown by Björn Lüssem et al., the drain current $I_{DS}$ of the transistor can be calculated as follows [29]:

(1) $$g_{m}=\frac{\partial I_{DS}}{\partial V_{GS}}$$

(2) $$I_{DS}=\frac{GV_{P}}{\frac{E_{0}}{kt}+1}\cdot \left[{\left(1-\frac{V_{G}-V_{D}}{V_{P}}\right)}^{\frac{E_{0}}{kt}+1}-{\left(1-\frac{V_{G}}{V_{P}}\right)}^{\frac{E_{0}}{kt}+1}\right]\;V_{D}<V_{D,sat}$$

(3) $$I_{DS}=\frac{GV_{P}}{\frac{E_{0}}{kt}+1}\cdot \left[{\left(1-\frac{V_{G}}{V_{P}}\right)}^{\frac{E_{0}}{kt}+1}\right]\;V_{D}>V_{D,sat}$$

(4) $$V_{P}=\frac{qp_{0}h}{C^{*}}$$

where $G=\frac{q\mu_{0}p_{0}Wh}{L}$ stands for the channel conductance at zero-field, and $V_{P}$ represents the pinch-off voltage at which the PEDOT:PSS layer is fully depleted at the drain. Once the channel is fully depleted at the drain, the drain current saturates. Thus, this drain voltage is referred to as the saturation voltage $V_{D,sat}=V_{G}-V_{P}$. $C^{*}$ is the specific volumetric capacitance of the channel. W, L, and h, are the channel dimensions of width, length, and thickness; $\mu_{0}$ is the hole’s mobility; $p_{0}$ is the zero-field hole concentration; k is Boltzmann’s constant; t is temperature; and $E_{0}$ is the disorder parameter describing the energetic width of the tail of the density of states. This equation shows that the capacitance of a gel electrolyte is a key parameter for the performance of OECTs.

As Figure 1d describes, in a classical electrical double-layer (EDL) model based on Gouy–Chapman–Stern (G-C-S) theory, the electrolyte can be divided into an inner layer or a Helmholtz layer (also known as the Stern layer), which is not accessible to the ionic species, and a diffuse layer, accounting for the inhomogeneous ion distributions [30,31,32]. By combining Equations (1), (2), and (4), we can derive that transconductance ($g_{m,\;max}$) is influenced by capacitance ($C^{*}$). In addition, the capacitance of a hydrogel electrolyte is affected by the electrical double-layer (EDL) effect. Therefore, our EDL-enhanced model systematically analyzes the key parameters that affect the electrolyte capacitance, which, in turn, further impacts the performance of OECTs.

Then, as shown in Figure 1e, the cation concentration distribution (at a voltage of 1 V, an ion concentration of 10^16^ cm^−3^, and a scan rate of 10 V/s) is simulated using the technology computer-aided design (TCAD) system. Figure 1f demonstrates the vertical cation concentration distribution derived from Figure 1e, revealing an exponential decrease in ion concentration, demonstrating the EDL effect in the gel electrolyte.

As the capacitance of the gel electrolyte consists of Helmholtz layer capacitance and diffusion layer capacitance in series, it can be seen that $\frac{1}{C^{*}}=\frac{1}{C_{H}}+\frac{1}{C_{D}}$ [30], where $C_{H}$ is the Helmholtz layer capacitance and $C_{D}$ is the diffusion layer capacitance. The GC theory predicts that the capacitance of the double layer is given by the following [33]:

(5) $$C_{D}=\varepsilon_{0}\varepsilon_{r}\kappa cosh\frac{\varphi }{2}$$

(6) $$\kappa =\frac{1}{\lambda_{D}}=\sqrt{\frac{2Z^{2}F^{2}c}{\varepsilon_{0}\varepsilon_{r}RT}}$$

(7) $$C_{H}\propto \frac{\varepsilon_{0}\varepsilon_{r}}{d}$$

where $\varepsilon_{0}$ is the permittivity of the free space; $\varepsilon_{r}$ is the dielectric constant or relative permittivity; $\lambda_{D}$ is the Debye length, c is the ion concentration, and F is Faraday‘s constant. $\varphi =\frac{1}{k_{B}T}Z\Psi$ is the reduced local electrical potential, k is the Boltzmann constant, T is temperature, Ψ is the electrostatic potential, Z is ion valence, and d is the diameter of an ion. Theoretical analysis derived from the above equation demonstrates that the capacitance of the gel electrolyte exhibits a direct dependence on three key parameters: ionic concentration, effective electrostatic potential, and scan rate. This fundamental relationship suggests a promising strategy for performance optimization in OECTs through the precise modulation of these operational parameters.

### 2.2. Simulation of Ion Distribution and Capacitance Under Different Conditions

**Figure 2:**
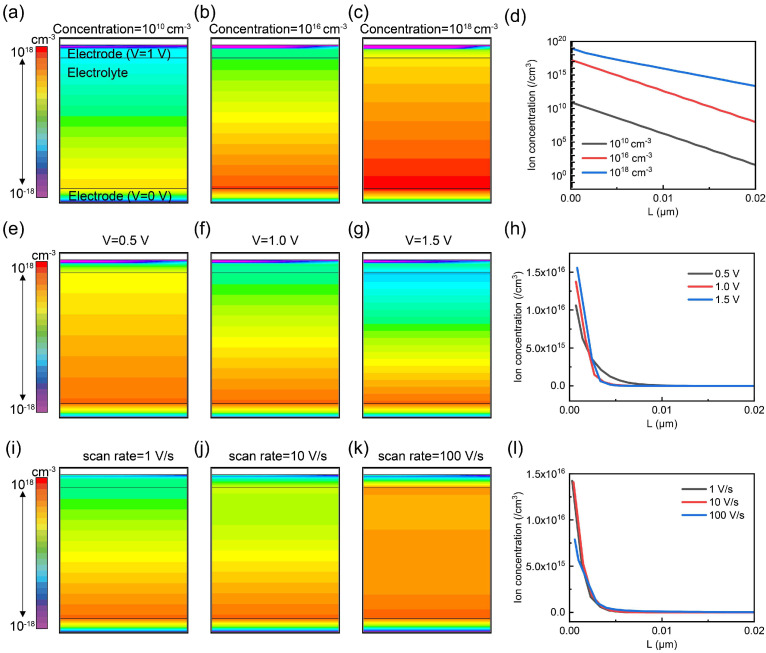
Ion distribution schematic of the devices. (**a**–**c**) Schematic of the ion densities of three devices at concentrations of 10^10^ cm^−3^, 10^16^ cm^−3^, and 10^18^ cm^−3^, respectively. (**d**) Ion distribution data comparison of three devices under different ion concentrations. (**e**–**g**) Schematic of the ion densities of three devices at voltages of 0.5 V, 1.0 V, and 1.5 V, respectively. (**h**) Ion distribution data comparison of three devices under different voltages. (**i**–**k**) Schematic of the ion densities of three devices at scan rates of 1 V/s, 10 V/s, and 100 V/s, respectively. (**l**) Ion distribution data comparison of three devices under different scan rates.

Since the capacitance of gel electrolytes is highly dependent on the ionic concentration, electrochemical potential, and modulation frequency, we designed a comprehensive series of simulations to systematically analyze the electric characteristics of OECT through the Silvaco TCAD 2018 software. Figure 2 presents the simulated ion distribution of the gel electrolyte under varying conditions. In Figure 2a–c, a voltage of 1 V is applied to the upper electrode, while the bottom electrode is fixed at 0 V, and the ion concentration within the device is set to 10^10^ cm^−3^, 10^16^ cm^−3^, and 10^18^ cm^−3^, respectively. As shown in Figure 2d, an increase in the initial ion concentration of the gel electrolyte results in a proportional increase in the ion density at the electrolyte/electrode interface, which directly affects the device’s electrical properties. Then, Figure 2e–g illustrate the influence of the applied voltages (0.5 V, 1.0 V, 1.5 V) on ion distribution within the gel electrolyte. As the applied voltage increases, the migration of ions towards the electrolyte/electrode interface is significantly accelerated, resulting in the ion concentration experiencing a remarkable increase, from 10^16^ cm^−3^ to 1.5 × 10^16^ cm^−3^, as vividly depicted in Figure 2h. This observation aligns well with the expected behavior of ion accumulation under external electric field modulation, further confirming the direct correlation between applied voltage and ion distribution in the electrolyte.

Additionally, the effect of scan rates on ion distribution is evaluated in Figure 2i–k. A lower scanning rate provides ample time for ions to migrate within the electrolyte. This enhanced time availability allows the ions to move more efficiently, guaranteeing that they have sufficient time to move. Therefore, as shown in Figure 2l, the ion concentration at the electrolyte/electrode interface experiences a remarkable increase with a lower scan rate, rising from 7.5 × 10^15^ cm^−3^ to 1.5 × 10^16^ cm^−3^.

In summary, the migration of ions within the electrolyte hinges on multiple factors, including the initial ion concentration, the applied potential, and the scan rate during operation. A higher ion concentration, a higher voltage, and a lower scanning rate within a certain range are conducive to ion migration and accumulation. This is of great significance for the subsequent analysis, aimed at understanding the reasons behind the performance changes in OECTs employing gel electrolytes.

### 2.3. Experimental Analysis of Factors Influencing the Capacitance of Gel Electrolytes

**Figure 3:**
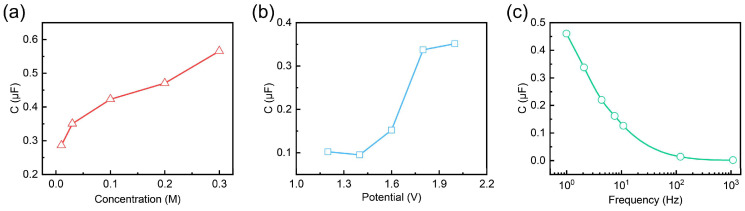
(**a**) Capacitance of the device under different concentrations. (**b**) Capacitance of the device under different potentials. (**c**) Capacitance of the device under different frequencies.

To complement the simulation results, we conducted experiments to investigate the effects of concentration, potential, and frequency on capacitance. As shown in Figure 3a, the capacitance ($C^{*}$) exhibits a positive correlation with the ion concentration within the gel electrolytes. As the ion concentration increases, the density of mobile charge carriers within the electrolyte rises, leading to a higher capacitance at the electrode/electrolyte interface. Figure 3b illustrates the effect of potential on capacitance. The capacitance remains stable at lower voltages but increases sharply beyond a certain threshold, due to the enhanced electric field promoting a stronger electrochemical double-layer effect. This suggests that a critical voltage is required to trigger significant ion accumulation at the interface, leading to greater charge storage capacity.

Figure 3c presents the frequency-dependent behavior of the capacitance. The capacitance decreases with increasing frequency. At low frequencies, ions in the gel electrolyte have sufficient time to migrate and contribute to charge accumulation at the interface, resulting in higher capacitance. In contrast, at higher frequencies, the rapid oscillation of the electric field limits the movement of ions, reducing their contribution to charge storage and leading to a lower capacitance. This frequency-dependent trend highlights the importance of ion mobility and relaxation time in determining the dynamic charge storage capability of the electrolyte.

Overall, these results suggest that optimizing the gel electrolyte’s concentration, potential range, and operational frequency is crucial for achieving the desired capacitance characteristics of gel electrolytes. Higher ion concentrations within gel electrolytes improve the charge storage efficiency, while an appropriate voltage range is essential for maximizing capacitance. Additionally, understanding the frequency response helps in designing devices that maintain stable capacitance across varying operating conditions. These insights provide a deeper understanding of the gel electrolyte’s role in electrochemical performance. In the subsequent work, we aim to verify the quantitative relationship between these parameters and their collective impact on the electrical performance of OECTs from both experimental data and theoretical formula calculation perspectives.

### 2.4. Experimental and Theoretical Calculation Analyses of Transfer Curve and Transconductance

**Figure 4:**
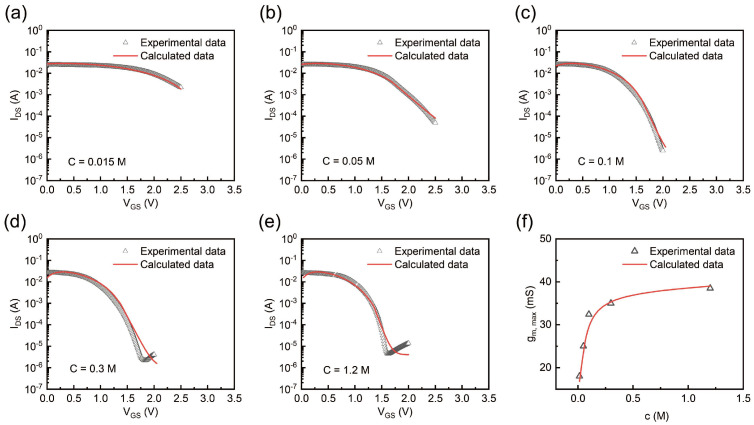
(**a**–**e**) Experimental and calculated transfer curves under different ion concentrations (0.015 M, 0.05 M, 0.1 M, 0.3 M, and 1.2 M, respectively). (**f**) Experimental data and calculated data of c − $g_{m,\;max}$.

To better understand the functional role of gel electrolytes in determining OECT properties, we designed and implemented a comprehensive series of experiments to systematically investigate the electric characteristics of OECT under different parameters. Additionally, the above models were applied for fitting the experimental data. These experimental and theoretical data supported the notion that gel electrolytes play a pivotal role in modulating these devices’ electrical properties. First, we delved into the influence of the ion concentration of the gel electrolyte on the device. Figure 4a–e illustrate the transfer characteristics at different ion concentrations. At a low ion concentration of 0.015 M (Figure 4a), the OECT struggled to completely turn off even at a high gate voltage, indicating insufficient ionic participation in the de-doping process of the PEDOT:PSS channel. As the concentration increased (Figure 4b–e), the $I_{DS}$ decreased more effectively with the gate voltage, and the threshold voltage shifted, indicating improved electrochemical gating efficiency. This trend highlights the critical role of the gel electrolyte’s composition and ion concentration in modulating device behavior.

Figure 4f reveals the relationship between the $g_{m,\;max}$ of OECTs and the ion concentration of the gel electrolyte. A theoretical analysis based on the above formula revealed that $g_{m}$ was proportional to $\sqrt{C}$. Therefore, the $g_{m,\;max}$ of OECTs increased with the ionic concentration under low-ionic-strength conditions. Then, as the EDL capacitance, which governs ion accumulation at the interface, saturated due to physical constraints or ion crowding in highly concentrated solutions, the $g_{m,\;max}$ of OECTs plateaued at a higher ion concentration of 1.2 M. These results emphasize that optimizing ionic concentration is crucial for maximizing transconductance while maintaining stable operation, providing valuable insights into tailoring OECT performance through electrolytes.

**Figure 5:**
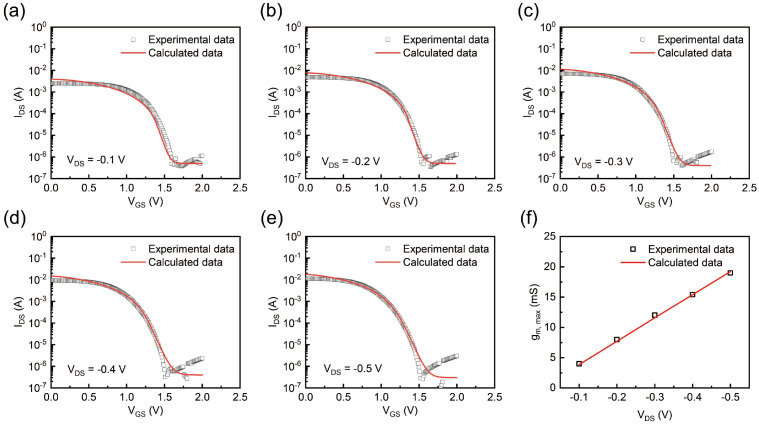
(**a**–**e**) Experimental and calculated transfer curves under different V_DS_ (−0.1 V, −0.2 V, −0.3 V, −0.4 V, and −0.5 V, respectively). (**f**) Experimental data and calculated data of V_DS_ −$g_{m,\;max}$.

As a three-terminal device architecture, the effective potential distribution across the gel electrolyte was jointly regulated by both the gate and drain voltages, establishing a dual-voltage-controlled electrochemical environment. Figure 5a–e present the transfer characteristics under varying drain voltages, from −0.1 V to −0.5 V, with a step of −0.1 V. As the drain voltage turned more and more negative, the effective potential difference between the gate and the channel increased. This enhanced potential difference drove more cations to infiltrate the channel layer and de-dope the PEDOT:PSS, thereby reducing the drain current [34]. As expected, the transconductance $g_{m,\;max}$ had a strong dependency with the applied drain potential V_DS_ (Figure 5f). Notably, as the drain voltage increased, $V_{G,\;max}$ shifted from the linear to the saturation region of the OECT, while the transconductance $g_{m,\;max}$ was independent of the drain voltage and no longer increased linearly [21]. This observation was validated by additional experiments, as shown in Figure S3, where $g_{m,\;max}$ gradually stabilized when V_D_ exceeded −1 V.

The observed relationship between V_DS_, $g_{m,\;max}$, and ion dynamics offered valuable insights into optimizing device operation through electrical bias control. By carefully tuning V_DS_, a higher $g_{m,\;max}$ can be achieved, leading to enhanced signal amplification and improved device performance, which is crucial for high-sensitivity and low-noise operation in the application of bioelectronic signal detection.

Additionally, the operation of OECT includes two processes: the ions’ transport to the semiconductor/electrolyte interface, and the ions’ injection into the channel where they undergo reactions with the semiconductor and regulate the conductivity of the channel [23]. Therefore, we suggest that the performance of OECTs is governed by the dynamics of ionic transport and the reactions occurring within the channel. A lower scan rate provides sufficient time for ions to transmit from the electrolyte to the channel and increases the possibility of a complexation reaction between Na^+^ and PSS⁻, which leads to the more effective modulation of the channel’s conductivity.

**Figure 6:**
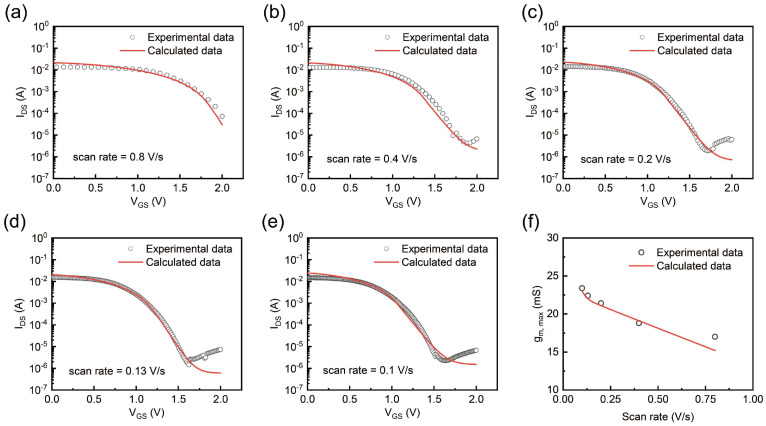
(**a**–**e**) Experimental and calculated transfer curves under different scan rates (0.8 V/s, 0.4 V/s, 0.2 V/s, 0.13 V/s, and 0.1 V/s, respectively). (**f**) Experimental data and calculated data of scan rate −$g_{m,\;max}$.

When exploring the factors influencing $I_{DS}$ and $g_{m,\;max}$, it is essential to consider scan rate S, which is related to ion transport dynamics [35]. To further investigate this relationship, we evaluate the device’s transfer characteristics across a range of scan rates. Figure 6a–e present the transfer curves at scan rates of 0.8 V/s, 0.4 V/s, 0.2 V/s, 0.13 V/s, and 0.1 V/s, respectively. In each curve, experimental data are compared with theoretical calculations. It is evident that, as the scan rate decreases, the OECT demonstrates improved performance, as indicated by a reduced turn-off current. This trend suggests that a slower scan rate offers enough time for more ions to flow from the electrolyte into the channel and undergo electrochemical reactions, while high scan rates cause ion response lags relative to the applied gate voltage, resulting in a low concentration of ions in the reactions. This observation reflects the importance of optimizing scan rates for maximizing OECT performance, particularly for applications requiring high precision and reliability in signal modulation.

Figure 6f highlights the dependence of $g_{m,\;max}$ on the scan rate. According to the calculation of the above formula, it can be deduced that g_m,max_ is inversely proportional to the scan rate. As the scan rate slows, $g_{m,\;max}$ increases significantly. Based on these experimental findings, we propose that the strategic modulation of the scan rate could significantly enhance the performance of OECTs. By carefully adjusting the scan rates, it is possible to achieve optimized ion transport dynamics, leading to optimized charge carrier injection, increased transconductance, and enhanced OECT performance.

## 3. Conclusions

This study refines the device model for OECTs by incorporating the EDL effect of gel electrolytes, addressing limitations in conventional models, and offering a more comprehensive framework for understanding their interactions with OECT performance. By systematically analyzing the influence of key parameters such as ion concentration, drain voltage, and scan rate on capacitance and peak transconductance through simulations, we establish a correlation between ionic transport dynamics and electrical performance. Theoretical calculation results and experimental validations confirm a strong correlation between hydrogel electrolytes and OECT performance. A higher ion concentration provides a greater supply of ions for electrochemical doping reactions, whereas an elevated drain voltage expedites the migration of ions towards the channel. Additionally, a reduced scan rate allows ample time for ions to transfer from the electrolyte to the channel, thereby facilitating the electrochemical doping reaction within the channel layer. By carefully tuning these parameters, OECT performance can be significantly optimized to meet the stringent requirements for high sensitivity, precision, and reliability in bioelectronic signal detection. These findings deepen our understanding of OECTs’ operating principles and provide guidance for designing high-performance devices in biosensing and bioelectronic applications.

## 4. Materials and Methods

### 4.1. Materials

The hydrogel was synthesized from polyvinyl alcohol (PVA (Alfa Aesar, Ward Hill, Massachusetts, USA)), acrylamide (AM (Sigma-Aldrich, Saint Louis, MO, USA)), 2-Hydroxy-4′-(2-Hydroxyethoxy)-2-Methylphenylacetone (photo initiator (Aladdin, Chicago, IL, USA)), (N,N)-Methylenebis-acrylamide (MBA (Sigma-Aldrich, Saint Louis, MO, USA)), glycerol (Gly (Aladdin, Chicago, IL, USA)), ethylene glycol (EG (Sigma-Aldrich, Saint Louis, MO, USA)), deionized water, and sodium chloride (NaCl (Sigma-Aldrich, Saint Louis, MO, USA)). Among these, the monomer AM underwent crosslinking with the crosslinker MBA under the photoinitiator, forming PAAm, which, together with PVA, formed a double-network hydrogel. The main role of EG and Gly was to enhance the water retention of the hydrogel, while Na^+^ served as free-moving ions in the gel-based electrolyte. The semiconductor channel used PEDOT:PSS (Clevios PH1000 (Heraeus, Hanau, Hesse, Germany)).

### 4.2. Experimental Method

The gate, source, and drain electrodes (5 nm Cr and 100 nm Au) were deposited on the substrate (polyethylene naphthalate, PEN) via a thermal evaporator(Shanghai Superconductor Technology Co.,Ltd., Shanghai, China). The channel length of the OECT was 400 μm, and the width was 1000 μm. Then, plasma treatment (50 W, 180 s) was applied to the channel region of the device with a mask to enhance its hydrophilicity, ensuring adhesion of the subsequently drop-cast PEDOT:PSS semiconductor solution to the channel area. Afterward, PEDOT:PSS was precisely drop-cast using a microsyringe and annealed at 90 °C for 1 h. Finally, the hydrogel electrolyte was drop-cast onto the gate and channel regions and then cured under UV light for one minute, completing the fabrication of the OECTs.

The experimental characteristics were measured using a semiconductor device analyzer (PDA, FS-Pro(Primarius Technologies, Shanghai, China)). The three probes of the PDA were in contact with the three electrodes of the OECT. A V_DS_ was applied between the source electrode and drain electrode, while the gate voltage was used to modulate the current in the source–drain channel, measuring the transfer characteristics of the OECT.

### 4.3. Simulation Method

The device structure and performance simulation was carried out using the Atlas module in Silvaco TCAD 2018 software(Silvaco International, Santa Clara, California, USA), following the procedure outlined below. First, the mesh must be defined. The mesh density should be adjusted according to the specific simulation requirements, ensuring a finer mesh at the electrode/electrolyte interface. If the mesh is too coarse, the calculation may fail to converge. Next, electrodes and material parameters need to be specified. For the electrolyte, we used custom parameters, with the dielectric constant and band gap set to values similar to those of the hydrogel used in the experiment, and incorporated ions to closely simulate its electrolyte characteristics. Subsequently, appropriate numerical models (e.g., model fermi) and iteration steps are selected for the simulation process. We selected the Fermi model because it defines that charge carriers in the initial state follow a Fermi distribution, which is widely adopted in simulations. Finally, an appropriate mathematical method (e.g., method Newton) is employed. We selected the Newton method because it is commonly used in finite-element simulations to achieve numerical convergence during the solving process. If the solution converges, the simulation results are analyzed to assess their consistency with experimental data. If convergence is not achieved, adjustments to the mesh density or step size may be required.

## Data Availability

The original contributions presented in this study are included in the article/Supplementary Materials. Further inquiries can be directed to the corresponding author(s).

## Supplementary Materials

The following supporting information can be downloaded at https://www.mdpi.com/article/10.3390/gels11030202/s1: Figure S1: (a) Schematic diagram of the operation mechanism of a FET (b) Schematic diagram of the operation mechanism of an OECT (c) Schematic diagram of the electrochemical reaction process of the OECT based on PEDOT:PSS; Figure S2: The preparation process of the dual network hydrogel electrolyte; and Figure S3: (a) Transfer curves under different VDS (−0.2V, −0.4 V, −0.6 V, −0.8 V, −1.0 V and −1.2 V, respectively) (b) Transconductance curves under different V_DS_ (c) V_DS_ –$g_{m,\;max}$ relationship diagram.

## References

[1] Yamada S., Toshiyoshi H. (2023). A biodegradable ionic gel for stretchable ionics. Sens. Actuators A-Phys..

[2] Tavares F.C., Cholant C.M., Kohlrausch E.C., Bolzan G.R., Goncalves P.F.B., Gil E.S., Khan S., Dupont J., Avellaneda C.O., Santos M.J.L. (2023). Ionic Liquid Boosted Conductivity of Biopolymer Gel Electrolyte. J. Electrochem. Soc..

[3] Kuddushi M., Xu B.B., Malek N., Zhang X. (2024). Review of ionic liquid and ionogel-based biomaterials for advanced drug delivery. Adv. Colloid Interface Sci..

[4] Li H., Hao Z., Zhang S., Li B., Wang Y., Wu X., Hu Y., Chen R., Chen T., Li J. (2023). “Smart” Stimuli-responsive Injectable Gels for Bone Tissue Engineering Application. Macromol. Biosci..

[5] Hyun J.E., Lim T., Kim S.H., Lee J.H. (2024). Wearable ion gel based pressure sensor with high sensitivity and ultra-wide sensing range for human motion detection. Chem. Eng. J..

[6] Lu M., Shen L., Su H., Li B., Wang L., Yu W.W. (2025). Highly ionic conductive, elastic, and biocompatible double-network composite gel for epidermal biopotential monitoring and wearable sensing. J. Colloid Interface Sci..

[7] Azimi M., Subramanian A., Fan J., Soavi F., Cicoira F. (2023). Electrical and mechanical stability of flexible, organic electrolyte-gated transistors based on iongel and hydrogels. J. Mater. Chem. C.

[8] Lee C.M., Kim Y., Kim W., Lee E., Lee E.K. (2024). High-Performance Synaptic Devices Based on Cross-linked Organic Electrochemical Transistors with Dual Ion Gel. Adv. Funct. Mater..

[9] Ren G., He S., Zhang Y., Zhu C., Gong Z., Wang K., Zhang L., Li Z., Lu G., Yu H.-D. (2023). Assessment of the Testing Methods for Evaluating the Performance of Organic Electrochemical Transistors. ACS Appl. Electron. Mater..

[10] Ghittorelli M., Lingstedt L., Romele P., Craciun N.I., Kovacs-Vajna Z.M., Blom P.W.M., Torricelli F. (2018). High-sensitivity ion detection at low voltages with current-driven organic electrochemical transistors. Nat. Commun..

[11] Yang A., Song J., Liu H., Zhao Z., Li L., Yan F. (2023). Wearable Organic Electrochemical Transistor Array for Skin-Surface Electrocardiogram Mapping Above a Human Heart. Adv. Funct. Mater..

[12] Romele P., Gkoupidenis P., Koutsouras D.A., Lieberth K., Kovacs-Vajna Z.M., Blom P.W.M., Torricelli F. (2020). Multiscale real time and high sensitivity ion detection with complementary organic electrochemical transistors amplifier. Nat. Commun..

[13] van De Burgt Y., Melianas A., Keene S.T., Malliaras G., Salleo A. (2018). Organic electronics for neuromorphic computing. Nat. Electron..

[14] Ji X., Paulsen B.D., Chik G.K.K., Wu R., Yin Y., Chan P.K.L., Rivnay J. (2021). Mimicking associative learning using an ion-trapping non-volatile synaptic organic electrochemical transistor. Nat. Commun..

[15] Azimi M., Kim C.-h., Fan J., Cicoira F. (2023). Effect of ionic conductivity of electrolyte on printed planar and vertical organic electrochemical transistors. Faraday Discuss..

[16] Bernards D.A., Malliaras G.G. (2007). Steady-state and transient behavior of organic electrochemical transistors. Adv. Funct. Mater..

[17] Romele P., Ghittorelli M., Kovacs-Vajna Z.M., Torricelli F. (2019). Ion buffering and interface charge enable high performance electronics with organic electrochemical transistors. Nat. Commun..

[18] Skowrons M., Dahal D., Paudel P.R., Luessem B. (2024). Depletion Type Organic Electrochemical Transistors and the Gradual Channel Approximation. Adv. Funct. Mater..

[19] Wang X., Shapiro B., Smela E. (2009). Development of a Model for Charge Transport in Conjugated Polymers. J. Phys. Chem. C.

[20] Kaphle V., Paudel P.R., Dahal D., Krishnan R.K.R., Lussem B. (2020). Finding the equilibrium of organic electrochemical transistors. Nat. Commun..

[21] Paudel P.R., Kaphle V., Dahal D., Radha Krishnan R.K., Lussem B. (2021). Tuning the Transconductance of Organic Electrochemical Transistors. Adv. Funct. Mater..

[22] Weissbach A., Cucchi M., Tseng H., Leo K., Kleemann H. (2023). Unraveling the Electrochemical Electrode Coupling in Integrated Organic Electrochemical Transistors. Adv. Funct. Mater..

[23] Rivnay J., Inal S., Salleo A., Owens R.M., Berggren M., Malliaras G.G. (2018). Organic electrochemical transistors. Nat. Rev. Mater..

[24] Khodagholy D., Rivnay J., Sessolo M., Gurfinkel M., Leleux P., Jimison L.H., Stavrinidou E., Herve T., Sanaur S., Owens R.M. (2013). High transconductance organic electrochemical transistors. Nat. Commun..

[25] Rivnay J., Leleux P., Sessolo M., Khodagholy D., Herve T., Fiocchi M., Malliaras G.G. (2013). Organic Electrochemical Transistors with Maximum Transconductance at Zero Gate Bias. Adv. Mater..

[26] Kaphle V., Liu S., Al-Shadeedi A., Keum C.-M., Lussem B. (2016). Contact Resistance Effects in Highly Doped Organic Electrochemical Transistors. Adv. Mater..

[27] Owens R.M., Malliaras G.G. (2010). Organic Electronics at the Interface with Biology. MRS Bull..

[28] Tybrandt K., Zozoulenko I.V., Berggren M. (2017). Chemical potential-electric double layer coupling in conjugated polymer-polyelectrolyte blends. Sci. Adv..

[29] Friedlein J.T., Shaheen S.E., Malliaras G.G., McLeod R.R. (2015). Optical Measurements Revealing Nonuniform Hole Mobility in Organic Electrochemical Transistors. Adv. Electron. Mater..

[30] Wu J. (2022). Understanding the Electric Double-Layer Structure, Capacitance, and Charging Dynamics. Chem. Rev..

[31] Le J.-B., Fan Q.-Y., Li J.-Q., Cheng J. (2020). Molecular origin of negative component of Helmholtz capacitance at electrified Pt(111)/water interface. Sci. Adv..

[32] Allagui A., Benaoum H., Olendski O. (2021). On the Gouy-Chapman-Stern model of the electrical double-layer structure with a generalized Boltzmann factor. Phys. A-Stat. Mech. Its Appl..

[33] Wang X., Liu K., Wu J. (2021). Demystifying the Stern layer at a metal-electrolyte interface: Local dielectric constant, specific ion adsorption, and partial charge transfer. J. Chem. Phys..

[34] Buth F., Kumar D., Stutzmann M., Garrido J.A. (2011). Electrolyte-gated organic field-effect transistors for sensing applications. Appl. Phys. Lett..

[35] Liang X., Luo Y., Pei Y., Wang M., Liu C. (2022). Multimode transistors and neural networks based on ion-dynamic capacitance. Nat. Electron..

