# Supplementary material for: Revealing the Impact of Gel Electrolytes on the Performance of Organic Electrochemical Transistors
Source: Gels. 2025 Mar 14;11(3):202. doi: 10.3390/gels11030202 (PMC11942148; doi:10.3390/gels11030202)
Supplement: Supplementary file 1 [file gels-11-00202-s001.zip › gels-3484663-supplementary.pdf]

Supporting Information

Revealing the Impact of Gel Electrolytes on the Performance of Organic Electrochemical Transistors

Mancheng Li [1], Xiaoci Liang [1], Chuan Liu [1], and Songjia Han [2,*]

1 State Key Laboratory of Optoelectronic Materials and Technologies and Guangdong Province Key Laboratory of Display Material and Technology, School of Electronics and Information Technology, Sun Yat-Sen Univer-sity, Guangzhou 510275, China;

2 College of Electronic Engineering, College of Artificial Intelligence, South China Agricultural University, Guangzhou 510642, China;

* Correspondence: hansongjia@scau.edu.cn

Figure S1

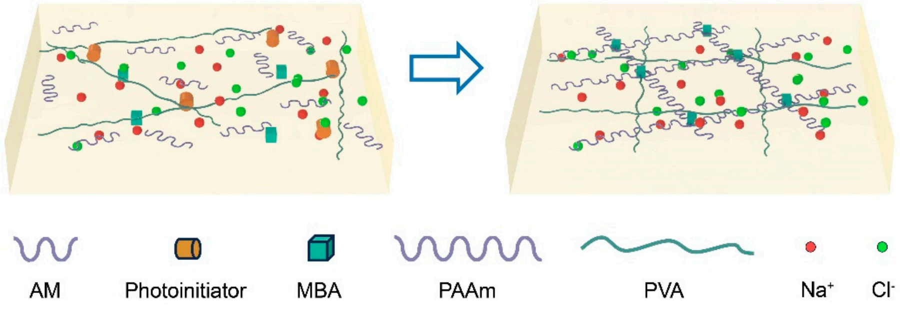

Figure S1. The preparation process of the dual network hydrogel electrolyte.
